# Family History of Diabetes and the Effectiveness of Lifestyle Intervention on Insulin Secretion and Insulin Resistance in Chinese Individuals with Metabolic Syndrome

**DOI:** 10.1155/2021/8822702

**Published:** 2021-01-04

**Authors:** Haiqing Zhu, Xiaoping Chen, Bo Zhang, Wenying Yang, Xiaoyan Xing

**Affiliations:** ^1^Department of Endocrinology, Emergency General Hospital, Beijing, China; ^2^Department of Endocrinology, China-Japan Friendship Hospital, Beijing, China

## Abstract

**Aims:**

The current study aims to explore if a family history of diabetes can influence the efficiency of lifestyle intervention on insulin secretion and study the insulin resistance in Chinese men and women with metabolic syndrome in a cohort with a 2-year follow-up.

**Methods:**

151 individuals (90 individuals did not have a family history of diabetes (DMFH (-)) and 61 with a family history of diabetes (DMFH (+)) with metabolic syndrome participated in the lifestyle intervention program at baseline and finished with 1-year follow-up. 124 individuals have two-year follow-up data. A family history of diabetes was ascertained by self-report. Lifestyle interventions were individual sessions on lifestyle changes.

**Results:**

During the 1-year follow-up, Ln Insulinogenic index (Δ_baseline−1year_ = 0.29 ± 0.65, *P* = 0.001) and 30-min glucose (Δ_baseline−1year_ = −0.41 ± 1.71, *P* = 0.024) changed significantly in the DMFH(-) group; in the DMFH(+) group, Ln ISIm (Δ_baseline−1year_ = −0.22 ± 0.60, *P* = 0.022) and 30-min glucose (Δ_baseline−1year_ = 0.53 ± 1.89, *P* = 0.032) changed significantly, and there was no significant change of other parameters. The change of 30 min glucose during a 1-year intervention has shown a significant difference between the two groups (*P* = 0.002). During the 2 years intervention, Ln Insulinogenic index changed significantly in the DMFH(-) group (Δ_baseline−1year_ = 0.33 ± 0.66, *P* < 0.001 and Δ_baseline−2year_ = 0.43 ± 1.17, *P* = 0.034). Fasting insulin (Δ_baseline−2year_ = 2.95 ± 8.69, *P* = 0.034), 2 h insulin (Δ_baseline−2year_ = 23.75 ± 44.89, *P* = 0.002), Ln HOMA-B (Δ_baseline−2year_ = 0.43 ± 1.02, *P* = 0.009), Ln HOMA-IR (Δ_baseline−2year_ = 0.53 ± 1.04, *P* = 0.002), Ln ISIm (Δ_baseline−2year_ = 0.52 ± 0.95, *P* = 0.004), and Ln Insulinogenic index (Δ_baseline−2year_ = 0.66 ± 1.18, *P* = 0.047) changed significantly after 2 years of intervention, compared to the baseline in the DMFH(+) group. The change of Ln ISIm (*P* = 0.023), fasting (*P* = 0.030), and 2 h insulin (*P* = 0.007) during the 2-year intervention has shown a significant difference between the two groups. Family history of diabetes was related with a 0.500 unit increase in 2-year ISIm (*P* = 0.020) modified by lifestyle intervention adjusted for age, baseline BMI, sex, and baseline waist circumference and a 0.476 unit increase in 2-year ISIm (*P* = 0.027) with extra adjustment for weight change.

**Conclusions:**

Patients with a family history of diabetes benefit more from lifestyle intervention in regard to insulin resistance than those without a family history of diabetes adjusting for age, baseline BMI, sex, baseline waist circumference, and weight change.

## 1. Introduction

Metabolic syndrome (MetS) is a collection of obesity, hypertension, dyslipidemia, and glucose intolerance and significantly increases the risk of type 2 diabetes mellitus (T2DM). MetS is characterized by insulin resistance and hyperinsulinemia, which lead to the deterioration of *β* cell function [1].

Lifestyle interventions are promising strategies to stop or delay the occurrence of T2DM [2]. Both genetic factors and adherence to lifestyle management influence the efficiency of lifestyle interventions. A family history of diabetes is known as one of the major risk factors for T2DM [3, 4]. Family history demonstrates the effects of genetic factors, clustered family lifestyle factors, and the relation between the features stated earlier [5]. Family history of diabetes also influences insulin resistance and insulin secretion, in the Chinese population [6]. The Finnish Diabetes Prevention Study (DPS) found that patients with reduced glucose tolerance and a family history of diabetes had a significant weight loss and decrease in two-hour plasma glucose relative to individuals without a family history of diabetes in the 1^st^ year of lifestyle intervention [7]. Previous reports documented that lifestyle intervention might be much prominent in the descendants of patients with T2DM compared to those without a family history of diabetes, with higher insulin sensitivity index in response to physical activity intervention, representing that insulin sensitivity is extremely controlled by exercise in patients with a family history of T2DM [8, 9].

The purpose of the current study is to evaluate if a patient family history of diabetes is related to the efficiency of lifestyle intervention on insulin resistance and insulin secretion in a cohort of metabolic syndrome with 2-year follow-up.

## 2. Subjects and Methods

### 2.1. Study Participants

Study participants were enrolled primarily from the outpatient department of endocrinology of China-Japan Friendship Hospital from June 2010 to May 2011. The selection criteria were as follows: (1) ages 30-70 years; (2) understand the whole process of the program, voluntarily participate, and sign an informed consent form; and (3) central obesity (female: waist circumference > 80 cm; male: waist circumference > 90 cm) accompanied by at least two of the following items:(1) 1.7 mmol/L < TG < 5.6 mmol/L and TC < 7.0 mmol/L; (2) 6.1 mmol/L ≤ fasting blood glucose (FPG) < 7.0 mmol/L; newly diagnosed diabetes mellitus, but FPG should be ≤ 8.0 mmol/L; (3) 130 mmHg ≤ systolic pressure < 160 mmHg, and/or 85 mmHg ≤ diastolic pressure < 100 mmHg, or received antihypertensive therapy; (4) HDL − C < 1.04 mmol/L for men, or <1.29 mmol/L for women. The exclusion criteria were as follows: (1) pregnant women and women who have an intention for pregnancy or breastfeeding; (2) patients who cannot cooperate; (3) patients with diastolic blood pressure ≥ 100 mmHg and/or systolic blood pressure ≥ 160 mmHg; (4) severe heart disease such as frequent angina pectoris or coronary artery bypass grafting or percutaneous coronary intervention, cardiac insufficiency, cardiac enlargement, myocardial infarction, and severe arrhythmia; (5) patients with stroke and transient ischemic attack; (6) patients with abnormal liver function, ALT, and AST more than 2 times of the normal upper limit; (7) patients with impaired renal function: serum creatinine, female ≥ 133 *μ*mol/L, male ≥ 135 *μ*mol/L; (8) patients with chronic gastrointestinal diseases; (9) patients with acute or chronic infections, malignant tumors, mental system diseases, and drug or alcohol addiction; and (10) patients who insist on using weight loss drugs. The exit criteria were as follows: (1) patient withdraws the informed consent form at any stage during the follow-up; (2) unable to cooperate and not following up on time; (3) pregnancy; (4) TG ≥ 5. 65 mmol/L, and/or TC ≥ 7. 0 mmol/L; (5) diastolic blood pressure > 100 mmHg and/or systolic blood pressure > 160 mmHg; (6) FPG > 11.0 mmol/L; and (7) occurrence of cardiovascular events. Finally, a total of 151 subjects (including 98 women and 53 men) were enrolled. All of the 151 patients finished a 1-year intervention, 124 of them finished a 2-year intervention, and 27 patients dropped out for the loss of follow-up or withdrawing.

### 2.2. Ethics Statement

The study protocol was approved by the ethics committee of the China-Japan Friendship Hospital (Beijing, China) and conducted in accordance with the Declaration of Helsinki II. We obtained written informed consent from each participant.

### 2.3. Study Design

A brief information was given to the subjects on how to reach the goals of the intervention: (1) among the patients with BMI (kg/m^2^) ≥ 24, decreasing 5–10% of initial body weight, and between patients with BMI < 24, request no weight loss; (2) <30% of energy derived from total fat consumption; (3) 55–65% of energy result from carbohydrate consumption, avoidance/decrease of refined carbohydrates, and white sugars; (4) 20–30 g fiber consumption/day, such as brown rice, whole grains, corn, fruits, and vegetables; and (5) doing modest or forceful physical activity for at least half an hour a day. The participants received monthly face-to-face sessions with study physicians. A detailed program was offered annually; in other monthly sessions, physicians only checked patients' weight, waist circumference and blood pressure, and gave them general oral evidence on the awareness of diabetes, dietary modification, and rising physical activity.

At baseline and at every annual visit, all participants completed a questionnaire about medical history and underwent a medical examination and an oral glucose tolerance examination.

### 2.4. Definitions

Diabetes was identified according to the 1999 World Health Organization (WHO) criteria of fasting plasma glucose (FPG) ≥ 7.0 mmol/L, 2 − h postprandial plasma glucose (2 − h PPG) ≥ 11.1 mmol/L, or a self-reported history of diabetes. Prediabetes was defined as FPG ≥ 6.1 and < 7.0 mmol/L and/or 2 − h PPG ≥ 7.8 and < 11.1 mmol/L. According to the 2005 IDF consensus worldwide definition of the metabolic syndrome for Asians, the criteria of MetS must include central obesity (waist circumference > 90 cm for males and >80 cm for females), plus two or more of the following risk factors, i.e., low HDL cholesterol (males < 1.04 mmol/L and females < 1.29 mmol/L, or under treatment), high serum triglycerides (>1.7 mmol/L, or under treatment), increased blood pressure (≥130/85 mmHg or under treatment), and fasting blood glucose (≥5.6 mmol/L or under treatment) [10].

### 2.5. Clinical Information and Laboratory Measurements

#### 2.5.1. Sociodemographic Characteristics

Data were collected with a planned questionnaire through a face-to-face interview to assess general information, personal history, family history, and history of current illness. A family history of diabetes was self-reported by a questionnaire at baseline. For the patients who have even one of first-degree relatives with diabetes, the family history of diabetes was measured to be positive.

#### 2.5.2. Anthropometric Measurements

Subjects were examined for hip circumference (HC), weight, height, waist circumference (WC), and blood pressure. Height and body weight were measured by standard protocol. BMI was calculated as weight/height^2^ (kg/m^2^). The WC was the circumference of the waist at the horizontal line of the umbilicus measured in centimeters through a measuring tape, and the HC was the circumference of hips at the horizontal line of the anterior superior spine measured in centimeters using a measuring tape. The blood pressure values used were an average of three measurements, which were taken 2 min apart using a mercury sphygmomanometer.

#### 2.5.3. Laboratory Examination

Venous blood samples after 8-14 hours of fasting were obtained from subjects for the measurement of triglyceride (TG), fasting blood glucose, HbA1c, total cholesterol (TC), low-density lipoprotein cholesterol (LDL-C), and high-density lipoprotein cholesterol (HDL-C). Blood samples during a 75 g oral glucose tolerance test (OGTT) were collected at 0 min, 30 min, and 2 h. By radioimmunoassay, serum insulin was assessed. In order to test the *β*-cell function, homeostatic model tests for insulinogenic indices and *β*-cell function (HOMA-B) were measured [11, 12]. The Matsuda index (ISIm) and HOMA-insulin resistance (IR) index were used to evaluate insulin sensitivity [12, 13].

### 2.6. Statistical Analysis

Continuous data are described using 95% confidence intervals and means, while categorical variables are concluded using percentages and counts. Continuous variables that were not normally distributed, including HOMA-B, HOMA-IR, ISIm, and insulinogenic indices, were natural log-transformed before analyses. The independent-sample *t*-test and *χ*^2^-test were used to evaluate the differences between the groups with and without a family history of diabetes for baseline characteristics and changes in characteristics. The paired-samples *t*-tests were used to evaluate the changes from baseline to 1 year/2 year. The general linear models were used to measure family history-lifestyle interaction effect on changes in primary outcomes, and multivariate-adjusted models were performed including age, gender, baseline BMI, baseline waist circumference, and family history of diabetes at baseline (model 1). Weight loss at 1 year and 2 years based on model 1 (model 2) were further adjusted. All mentioned *P* values were nominal and 2-sided, and *P* < 0.05 was considered significant. All statistical analyses were performed using the SPSS statistical software version 20.0.

## 3. Results

### 3.1. Clinical Characteristics

Among the 151 participants, the DMFH(-) and DMFH(+) groups had 90 and 61 participants. Compared to those in the DMFH(-) group (DBP 86.99 ± 7.16 mmHg), participants in the DMFH(+) (DBP 83.82 ± 10.35 mmHg) group had lower diastolic blood pressure. There were no significant differences among other clinical parameters, as shown in Table 1.

### 3.2. Baseline/1-Year/2-Year Glucose Tolerance Characteristics in Subjects with and without Family History of Diabetes

According to the 1998 WHO criteria, the baseline proportion of normal glucose tolerance (NGT), prediabetes, and diabetes was 50.0% (45/9), 40.0% (36/90), and 10.0% (9/90) in the DMFH(-)group, and 55.7% (34/61), 27.9% (17/61), and 16.4% (10/61) in the DMFH(+) group, respectively. After 1-year lifestyle intervention, in the DMFH(-)group, 26.7% of the NGT participants developed into prediabetes or DM, 41.7% of the pre-DM patients turned into NGT, and 8.3% of them developed into DM. 22.2% of the DM patients turned into pre-DM. In the DMFH(+)group, 11.8% of the NGT participants developed into pre-diabetes or DM, 47.1% of the pre-DM patients turned into NGT, and 11.8% of them developed into DM. 50.0% of the DM patients turned into pre-DM, and 20% of them turned into NGT (Table 2). After a 2-year lifestyle intervention, in the DMFH(-) group, 25.0% of the NGT participants developed into pre-diabetes or DM, 46.7% of the pre-DM patients turned into NGT, and 13.3% of them developed into DM. 37.5% of the DM patients turned into pre-DM, and 37.5% of them turned into NGT. In the DMFH(+) group, 32.0% of the NGT participants developed into pre-diabetes or DM, 58.3% of the pre-DM patients turned into NGT, and 8.4% of them developed into DM. 55.6% of the DM patients turned into pre-DM, and none of them turned into NGT (Table 3).

### 3.3. Changes of Insulin Secretion and Insulin Resistance Indices after 1-Year/2-Year Lifestyle Intervention in Subjects with and without a Family History of Diabetes

Clinical characteristics related to glucose metabolism such as weight, fasting/30 min/2 h glucose, fasting/2 h insulin during OGTT, and indices such as HOMA-B, HOMA-IR, ISIm, and Insulinogenic index were compared separately in and between the two groups throughout the 2-year intervention. All of the 151 participants finished 1-year intervention; the above indices show no significant difference between the two groups at baseline, except for Ln ISIm, and the DMFH(+) group had lower ISIm compared to DMFH(-) (1.48 ± 0.47 vs. 1.68 ± 0.52, *P* = 0.045), which indicates lower insulin sensitivity. At the end of the 1^st^ year, the above parameters have shown no significant difference between the two groups. During the 1^st^ year intervention, Ln Insulinogenic index (Δ_baseline−1year_ = 0.29 ± 0.65, *P* = 0.001) and 30-min glucose (Δ_baseline−1year_ = −0.41 ± 1.71, *P* = 0.024) changed significantly in the DMFH(-) group; in the DMFH(+) group, Ln ISIm (Δ_baseline−1year_ = −0.22 ± 0.60, *P* = 0.022) and 30-min glucose (Δ_baseline−1year_ = 0.53 ± 1.89, *P* = 0.032) changed significantly, and there was no change reported among the other parameters. The change of 30 min glucose during 1-year intervention has shown a significant difference between the two groups (*P* = 0.002) (Table 4).

A total of 124 participants finished the 2-year intervention; at the end of the 2^nd^ year, the DMFH(+) group had lower Ln Homa-B (4.02 ± 0.88 vs. 4.43 ± 1.14, *P* = 0.039) and fasting insulin (7.15 ± 5.54 vs. 11.25 ± 9.99, *P* = 0.013) compared to the DMFH(-) group. During the 2-year intervention, the Ln Insulinogenic index changed significantly in the DMFH(-) group (Δ_baseline−1year_ = 0.33 ± 0.66, *P* < 0.001 and Δ_baseline−2year_ = 0.43 ± 1.17, *P* = 0.034). Fasting insulin (Δ_baseline−2year_ = 2.95 ± 8.69, *P* = 0.034), 2 h insulin (Δ_baseline−2year_ = 23.75 ± 44.89, *P* = 0.002), Ln HOMA-B (Δ_baseline−2year_ = 0.43 ± 1.02, *P* = 0.009), Ln HOMA-IR (Δ_baseline−2year_ = 0.53 ± 1.04, *P* = 0.002), Ln ISIm (Δ_baseline−2year_ = −0.52 ± 0.95, *P* = 0.004), and Ln Insulinogenic index (Δ_baseline−2year_ = 0.66 ± 1.18, *P* = 0.047) changed significantly after 2 years of intervention compared to baseline in the DMFH(+) group. The change of Ln ISIm (*P* = 0.023), fasting (*P* = 0.030), and 2 h insulin (*P* = 0.007) during the 2-year intervention has shown a significant difference between the two groups (Table 5).

### 3.4. Associations of Family History of Diabetes with the Insulin Secretion-Related and Insulin Resistance-Related Indices after 1 or 2 Years of Lifestyle Intervention

The relationship between family history of diabetes and 1-year changes in HOMA-IR, HOMA-B, ISIm, or Insulinogenic index was not significantly changed by lifestyle intervention adjusting for age, baseline BMI, sex, baseline waist circumference in model 1, and with extra tuning for weight change in model 2. A family history of diabetes was related to a 0.500 unit increase in the 2-year ISIm (*P* = 0.020) in model 1 and a 0.476 unit increase in the 2-year ISIm (*P* = 0.027) in model 2. However, there was no significant association between family history of diabetes and 2-year changes in HOMA-IR, HOMA-B, or Insulinogenic index (Table 6).

## 4. Discussion

Nutrient excess and sedentary behaviors of our modern society are indications of metabolic syndrome, which significantly increases T2DM risk and gives a natural state of decreased insulin sensitivity and offers a common physiological *β*-cell challenge. Lifestyle intervention reports have shown that a diet/exercise regimen reduces IGT progression to T2DM by increasing insulin sensitivity and increasing insulin secretion. Lifestyle management is also the basis for the medical care of diabetes[14, 15]. In our study, we enrolled MetS patients with normal glucose, prediabetes, or mild newly onset diabetes and performed a 2-year lifestyle intervention to examine its effects on insulin secretion and insulin resistance.

Based on our 2-year longitudinal study, the results indicated that patients with a family of diabetes and metabolic syndrome would benefit more significantly from lifestyle intervention in regard to insulin resistance compared to those without a family history and not dependent on weight change. In patients without a family history of diabetes, the Insulinogenic index which indicated an early phase of insulin secretion continued to worsen throughout 2 years of lifestyle intervention; however, insulin resistance did not change significantly in the DMFH(-) group. This result indicated that in these patients, besides the traditional lifestyle intervention plan, the additional targeted method should be applied to ameliorate the early-phase insulin secretion, such as low glycemic index food, or *α*-glucosidase inhibitor. It is worthy to note that the discrepancy in insulin secretion and insulin resistance between different races, Asians appear to worse in early phase insulin secretion than Caucasians [16]. A previous study shown that T2DM in East Asians was characterized primarily by *β*-cell dysfunction, which was evident immediately after ingestion of glucose or meal, and less adiposity compared to that in Caucasians [17]. A study has shown that increased HbA_1_c was related to compromised early-phase insulin secretion relatively compared to the insulin resistance in Koreans which were at high risk of diabetes [18]. In patients with a family history of diabetes, the delightful change in 1 year was an improvement of ISIm, and this continued in the 2^nd^ year with improved HOMA-IR; however, HOMA-B and Insulinogenic index, which represented insulin secretion, worsen in the 2^nd^ year.

In the present study, intervention in lifestyle involved dietary improvements (intake of calories, fat, carbohydrates, fiber) and physical activity increases. Convincing research indicated that dietary variables controlled the sensitivity of insulin [19, 20]. Cohort and nutritional intervention studies suggested that people with greater genetic predisposition could prevent complex dietary habits which were more detrimental in the heterogeneity of a particular T2DM-related phenotype [21]. Furthermore, enriched exercise could also advance insulin sensitivity and glucose homeostasis [22]. In summary, we concluded that the combination of enhanced dietary intakes and physical activity could alter the impact of the diabetes family history on insulin sensitivity.

Family history not only represents the ramifications of numerous genetic variables, but also the family's clustered lifestyle variables. A previous study has shown that, despite having comparable recorded physical activities and exercise to that of individuals with no family history, a family history of T2DM was correlated with lower physical fitness. The same participants have reduced insulin secretion optimized for insulin resistance, despite increased BMI [23]. A previous study has shown that individuals without a family history of diabetes were more effective in reacting to lifestyle therapy about cardiometabolic assessments and glucose tolerance compared to the individuals with a family history of diabetes in a 1-year follow-up in a cohort of Finnish men at significant risk for T2DM [24]. However, in our study, the patients of the two groups have shown no significant difference in fasting glucose, 2 h glucose, or HbA_1_c, and those with a family history of diabetes were more effective in retorting to lifestyle therapy in terms of insulin resistance.

The family history-lifestyle intervention on changes in insulin resistance only becomes significant in 2 years. These findings were different from the findings reported in other long-term diet or lifestyle intervention trials such as the A TO Z Weight Loss Study and the DIRECT trial [25, 26]. In the A TO Z Weight Loss Study and DIRECT trial, the changes in insulin resistance weakened from 1 year to 2 years, comparatively due to reduced devotion to exercise and dietary and intervention after 1 year. However, in our study, both groups did not change significantly in body weight, especially in the DMFH(+) group, and the result has shown family history affected the ISIm after adjusting baseline BMI and weight change.

The current study has many significant findings. The present study was a 2-year longitudinal study, the findings were based on longitudinal measures of weight and glycemic markers, and its participants were suffering from metabolic syndrome including NGT, prediabetes, and diabetes. The most important and common feature was insulin resistance. We performed OGTT to get indices such as ISIm and Insulinogenic index in addition to HOMA-B and HOMA-IR, which were only based on fasting glucose and insulin to get a more comprehensive profile of *β*-cell function. At the same time, the present study gives insight into the various mechanisms that support family history affecting the effectiveness of lifestyle intervention.

This study also has limitations. First, at subsequent monthly visits, all the participants received written and general oral guidance on diabetes awareness, dietary change, and increased exercise, although no unique individualized programs were provided. We did not perform a questionnaire on the changes in the main exercise habits and dietary, 3-day 24-h food records to testify the result, and the level of our intervention. This kind of lifestyle intervention saved time and mimicked the most common lifestyle intervention in the clinical practice of outpatient departments in China, but it made the procedure less precise and targeted. Secondly, the comparatively small sample size may restrict the power of the analysis to detect much more moderate associations.

In conclusion, we reported a family history of diabetes was related with a 0.500 unit greater increase in 2-year ISIm (*P* = 0.020), modified by lifestyle intervention adjusting for age, baseline BMI, sex, and baseline waist circumference, and a 0.476 unit greater increase in 2-year ISIm (*P* = 0.027) with extra adjustment for weight change. These findings indicated that patients with a family history of diabetes were more effective in acting to lifestyle counselling with regard to insulin resistance than those without a family history of diabetes with additional adjustment for weight change. In this regard, it stresses the significance of a thorough examination of family history, in the risk assessment and development of more targeted therapeutic strategies for lifestyle interventions and diabetes prevention.

## Figures and Tables

**Table 1:** Baseline clinical characteristics of the participants based on the history of diabetes.

	DMFH- (*n* = 90)	DMFH+ (*n* = 61)	*P*
Age (year)	48.76 ± 8.95	48.93 ± 7.96	0.905
Male sex (*n*, %)	32 (35.6)	21 (34.4)	0.887
WC (cm)	92.96 ± 8.11	93.12 ± 9.09	0.908
Weight (kg)	74.67 ± 10.40	74.61 ± 10.03	0.972
WHR	0.90 ± 0.06	0.90 ± 0.05	0.886
Body mass index (kg/m^2^)	28.28 ± 3.34	28.34 ± 4.12	0.925
Systolic blood pressure (mmHg)	133.96 ± 12.01	132.05 ± 11.14	0.326
Diastolic blood pressure (mmHg)	86.99 ± 7.16	83.82 ± 10.35	0.028
Total cholesterol (mmol/L)	5.05 ± 1.04	4.84 ± 0.74	0.185
Triglyceride (mmol/L)	2.41 ± 1.23	2.10 ± 0.67	0.083
HDL-C (mmol/L)	1.17 ± 0.26	1.20 ± 0.25	0.554
LDL-C (mmol/L)	3.20 ± 1.12	3.30 ± 0.86	0.579
Fasting glucose (mmol/L)	5.62 ± 0.71	5.63 ± 0.78	0.980
30 min glucose (mmol/L)	9.89 ± 1.94	10.50 ± 2.46	0.091
2-h glucose (mmol/L)	7.70 ± 2.06	7.86 ± 2.88	0.696
Fasting insulin (mU/L)	9.63 ± 6.57	10.43 ± 6.81	0.483
30 min insulin (mU/L)	58.21 ± 39.45	62.13 ± 43.20	0.578
2-h insulin (mU/L)	49.23 ± 43.75	59.89 ± 50.34	0.182
HbA_1_C	5.45 ± 0.59	5.58 ± 0.74	0.214

**Table 2:** One-year glucose tolerance outcome in subjects with and without a family history of diabetes.

		DMFH- (*n* = 90)			DMFH+ (*n* = 61)		
		NGT	Pre-DM	DM	NGT	Pre-DM	DM
Baseline (*n*, %)		45 (50.0)	36 (40.0)	9 (10.0)	34 (55.7)	17 (27.9)	10 (16.4)
1year outcome	NGT (*n*, %)	33 (73.3)	15 (41.7)	0 (0.0)	30 (88.2)	8 (47.1)	2 (20.0)
	Pre-DM (*n*, %)	11 (24.5)	18 (50.0)	2 (22.2)	3 (8.9)	7 (41.1)	5 (50.0)
	DM (*n*, %)	1 (2.2)	3 (8.3)	7 (77.8)	1 (2.9)	2 (11.8)	3 (30.0)

**Table 3:** Two-year glucose tolerance outcome in subjects with and without a family history of diabetes.

		DMFH- (*n* = 78)			DMFH+ (*n* = 46)		
		NGT	Pre-DM	DM	NGT	Pre-DM	DM
Baseline (*n*, %)		40 (51.3)	30 (38.5)	8 (10.2)	25 (54.3)	12 (26.1)	9 (19.6)
2-year outcome	NGT (*n*, %)	30 (75.0)	14 (46.7)	3 (37.5)	17 (68.0)	7 (58.3)	0 (0.0)
	Pre-DM (*n*, %)	8 (20.0)	12 (40.0)	3 (37.5)	7 (28.0)	4 (33.3)	5 (55.6)
	DM (*n*, %)	2 (5.0)	4 (13.3)	2 (25.0)	1 (4.0)	1 (8.4)	4 (44.4)

**Table 4:** Baseline characteristics and 1-year changes in insulin secretion and insulin resistance according to the family history of type 2 diabetes.

	DMFH- (*n* = 90)				DMFH+ (*n* = 61)					
	Baseline	1 year	Change	*P*_Δbaseline−1year_^∗^	Baseline	1 year	Change	*P*_Δbaseline−1year_^∗^	*P*_ΔDMFH(−)VS(+)_^∗∗^	*P*_baseline DMFH(−)VS(+)_
Ln Homa-IR	0.70 ± 0.60	0.56 ± 0.61	0.13 ± 0.66	0.064	0.81 ± 0.58	0.66 ± 0.78	0.14 ± 0.74	0.183	0.986	0.296
Ln Homa-B	4.39 ± 0.73	4.31 ± 0.64	0.10 ± 0.75	0.233	4.46 ± 0.60	4.43 ± 0.70	0.06 ± 0.71	0.533	0.774	0.541
Ln Insulinogenic index	2.40 ± 0.83	2.14 ± 0.74	0.29 ± 0.65	0.001	2.27 ± 0.84	2.30 ± 0.90	0.05 ± 0.90	0.746	0.105	0.428
Ln ISIm	1.68 ± 0.52	1.76 ± 0.60	−0.08 ± 0.51	0.201	1.48 ± 0.47	1.63 ± 0.66	−0.22 ± 0.60	0.022	0.204	0.045
Weight (kg)	74.67 ± 10.40	72.7 ± 10.35	2.30 ± 11.59	0.084	74.61 ± 10.03	74.8 ± 9.01	0.67 ± 6.75	0.507	0.934	0.972
Fasting glucose (mmol/L)	5.62 ± 0.71	5.61 ± 0.89	0.01 ± 0.74	0.867	5.63 ± 0.78	5.56 ± 0.90	0.07 ± 0.82	0.530	0.680	0.980
30 min glucose (mmol/L)	9.89 ± 1.94	10.30 ± 2.11	−0.41 ± 1.71	0.024	10.50 ± 2.46	9.97 ± 2.25	0.53 ± 1.89	0.032	0.002	0.091
2-h glucose (mmol/L)	7.70 ± 2.06	7.95 ± 2.67	−0.25 ± 2.06	0.249	7.86 ± 2.88	7.48 ± 2.69	0.38 ± 2.38	0.219	0.085	0.696
Fasting insulin(mU/L)	9.63 ± 6.57	8.39 ± 5.35	1.27 ± 6.06	0.052	10.43 ± 6.81	10.13 ± 9.52	0.13 ± 7.22	0.897	0.313	0.483
2-h insulin(mU/L)	49.23 ± 43.75	46.09 ± 43.09	3.21 ± 33.78	0.375	59.89 ± 50.34	52.18 ± 45.93	8.28 ± 43.30	0.170	0.439	0.182

HOMA-B: the homeostasis model assessment for *β*-cell function; HOMA-IR: the homeostasis model assessment for insulin resistance; ISIm: Matsuda index; *P* < 0.05 is considered statistically significant. ^∗^*P* values for paired-samples *t*-tests for changes from baseline to 1 year. ^∗∗^*P* values for independent sample *t*-test for 1-year changes.

**Table 5:** Baseline characteristics/1-year/2-year changes in insulin secretion and insulin resistance based on the family history of type 2 diabetes.

	DMFH- (*n* =78)							DMFH+ (*n* = 46)									
	Baseline	1 year	2 years	change_Δbaseline−1year_	change_Δbaseline−2year_	*P*_Δbaseline−1year∗_	*P*_Δbaseline−2year∗_	Baseline	1 year	2 years	change_Δbaseline−1year_	change_Δbaseline−2year_	*P*_Δbaseline−1year∗_	*P*_Δbaseline−2year∗_	*P*_1yearΔDMFH(−)VS(+)∗∗_	*P*_2yearΔDMFH(−)VS(+)∗∗_	*P*_baseline DMFH(−)VS(+)_
Ln Homa-IR	0.69 ± 0.61	0.54 ± 0.63	0.58 ± 0.98	0.15 ± 0.69	0.13 ± 1.18	0.058	0.354	0.78 ± 0.61	0.69 ± 0.81	0.26 ± 0.87	0.06 ± 0.76	0.53 ± 1.04	0.595	0.002	0.524	0.070	0.449
Ln Homa-B	4.40 ± 0.75	4.30 ± 0.67	4.43 ± 1.14	0.13 ± 0.78	−0.01 ± 1.32	0.160	0.959	4.41 ± 0.56	4.40 ± 0.72	4.02 ± 0.88	0.02 ± 0.76	0.43 ± 1.02	0.836	0.009	0.500	0.065	0.941
Ln Insulinogenic index	2.39 ± 0.87	2.10 ± 0.75	1.98 ± 1.18	0.33 ± 0.66	0.43 ± 1.17	0.000	0.034	2.26 ± 0.91	2.22 ± 0.89	1.97 ± 0.98	0.10 ± 0.97	0.66 ± 1.18	0.575	0.047	0.181	0.521	0.513
Ln ISIm	1.70 ± 0.52	1.79 ± 0.61	1.78 ± 0.86	−0.11 ± 0.54	−0.04 ± 0.92	0.149	0.786	1.50 ± 0.45	1.62 ± 0.63	2.08 ± 0.80	−0.16 ± 0.59	−0.52 ± 0.95	0.130	0.004	0.650	0.023	0.075
Weight (kg)	75.10 ± 10.59	74.92 ± 11.21	72.80 ± 10.38	0.18 ± 7.05	2.24 ± 11.65	0.820	0.096	75.18 ± 9.24	75.60 ± 8.45	74.91 ± 9.08	−0.12 ± 4.80	0.73 ± 6.82	0.870	0.479	0.800	0.436	0.965
Fasting glucose (mmol/L)	5.59 ± 0.73	5.59 ± 0.90	5.45 ± 0.82	0.00 ± 0.77	0.13 ± 0.9	0.988	0.192	5.65 ± 0.80	5.64 ± 0.94	5.55 ± 0.85	0.01 ± 0.85	0.10 ± 0.86	0.932	0.430	0.950	0.845	0.647
30 min glucose (mmol/L)	9.92 ± 2.02	10.27 ± 2.13	9.88 ± 2.21	−0.35 ± 1.75	0.05 ± 2.32	0.080	0.858	10.56 ± 2.67	10.12 ± 2.39	10.01 ± 2.29	0.43 ± 1.86	0.55 ± 2.07	0.120	0.078	0.020	0.230	0.136
2-h glucose (mmol/L)	7.71 ± 2.00	8.02 ± 2.74	7.56 ± 2.11	−0.30 ± 2.15	0.13 ± 2.28	0.216	0.604	8.09 ± 3.00	7.61 ± 2.76	7.94 ± 2.82	0.48 ± 2.52	0.15 ± 2.42	0.207	0.675	0.070	0.969	0.407
Fasting insulin (mU/L)	9.66 ± 6.86	8.32 ± 5.58	11.25 ± 9.99	1.39 ± 6.37	−1.60 ± 11.71	0.058	0.239	10.11 ± 6.55	10.48 ± 10.6	7.15 ± 5.54	−0.69 ± 7.21	2.95 ± 8.69	0.545	0.034	0.109	0.030	0.730
2-h insulin (mU/L)	48.28 ± 43.63	45.76 ± 44.13	48.88 ± 43.31	2.55 ± 33.45	−0.46 ± 46.21	0.502	0.932	50.04 ± 43.3	50.59 ± 44.95	36.67 ± 40.45	6.53 ± 41.73	23.75 ± 44.89	0.329	0.002	0.576	0.007	0.419

HOMA-B: the homeostasis model assessment for *β*-cell function; HOMA-IR: the homeostasis model assessment for insulin resistance; ISIm: Matsuda index. *P* < 0.05 is considered statistically significant. ^∗^*P* values for paired-samples *t*-tests for changes from baseline to 1 year/2 years. ^∗∗^*P* values for independent sample *t*-test for 1-year/2-year changes.

**Table 6:** Effects of the family history of diabetes on changes in insulin secretion and insulin resistance in the response of lifestyle intervention.

	1-year outcome			2-year outcome		
	*β*	SE	*P*	*β*	SE	*P*
Model 1						
*Δ*HOMA-B	0.030	0.129	0.819	-0.406	0.238	0.091
*Δ*HOMA-IR	-0.013	0.123	0.918	-0.361	0.222	0.107
*Δ*Insulinogenic index	0.279	0.154	0.077	-0.244	0.342	0.479
*Δ*ISIm	0.148	0.112	0.190	0.500	0.210	0.020
Model 2						
*Δ*HOMA-B	0.030	0.130	0.816	-0.399	0.243	0.104
*Δ*HOMA-IR	-0.110	0.115	0.340	-0.344	0.225	0.130
*Δ*Insulinogenic index	0.276	0.155	0.078	-0.230	0.348	0.512
*Δ*ISIm	0.150	0.112	0.182	0.476	0.211	0.027

Adjusting for age, baseline BMI, sex, and baseline waist circumference in model 1 and with extra adjustment for weight change in model 2.

## Data Availability

The data used to support the findings of this study are available from the corresponding author upon request.
